# Thirty years after anorexia nervosa onset, serum neurofilament light chain protein concentration indicates neuronal injury

**DOI:** 10.1007/s00787-020-01657-7

**Published:** 2020-10-11

**Authors:** Elisabet Wentz, Sandra Rydberg Dobrescu, Lisa Dinkler, Carina Gillberg, Christopher Gillberg, Kaj Blennow, Maria Råstam, Henrik Zetterberg

**Affiliations:** 1 Department of Psychiatry and Neurochemistry, Institute of Neuroscience and Physiology, University of Gothenburg, Gothenburg, Sweden; 2 Högsbo Hospital, House B4, Tunnlandsgatan 2A, 421 38 Västra Frölunda, Sweden; 3 Gillberg Neuropsychiatry Centre, Institute of Neuroscience and Physiology, University of Gothenburg, Gothenburg, Sweden; 4 Department of Child and Adolescent Psychiatry, University of Glasgow, Glasgow, UK; 5 Clinical Neurochemistry Laboratory, Sahlgrenska University Hospital, Mölndal, Sweden; 6 Department of Clinical Sciences Lund, Child and Adolescent Psychiatry, Lund University, Lund, Sweden; 7 Department of Neurodegenerative Disease, UCL Institute of Neurology, London, UK; 8 UK Dementia Research Institute at UCL, London, UK

**Keywords:** Anorexia nervosa, Long-term follow-up, Neurofilament light chain protein, Neurodegenerative biomarker

## Abstract

Little is known about the long-term consequences of anorexia nervosa (AN) in terms of possible brain neuronal injury. We aimed at investigating whether women with adolescent-onset AN exhibit increased serum levels of neurofilament light chain protein (NfL), a biomarker for neuronal injury, compared with matched controls at 30-year follow-up. Blood samples were collected from 34 women with adolescent-onset AN and 38 matched healthy comparison women (COMP), at a mean age of 44 years (range 38–48 years). NfL was measured in serum using the in-house single molecule array (Simoa) method. The individuals were asked whether they or their parents had been diagnosed with dementia. The Swedish National Patient Register was searched for diagnoses related to dementia. Serum NfL concentrations were significantly higher in the AN group (AN 27.7 pg/ml; COMP 19.0 pg/ml; *p* = 0.041). When individuals with medical/neurological disorders in the AN and COMP groups were excluded, there was a statistically non-significant trend towards higher concentrations in the AN group (AN 27.4 pg/ml; COMP 18.8 pg/ml; *p* = 0.060). None of the participants had been diagnosed with dementia. There was no significant correlation between serum NfL and AN duration (*r* = 0.15). There was a moderate negative correlation between the serum NfL concentration and the current BMI in the AN group (*r* = 0.44). This is the first time that serum NfL has been assessed in middle-aged women with a history of adolescent-onset AN. The results suggest that there might be increased axonal degeneration as a sequel of AN. Individuals remaining underweight had higher serum NfL concentrations than those with a normal/high BMI. Additional studies are needed to confirm increased serum NfL concentrations in individuals recovered from AN. There is a need for further study of axonal degeneration as a consequence of AN.

## Introduction

Anorexia nervosa (AN) is a severe psychiatric disorder that mainly affects adolescent and young adult females and has its peak onset in adolescence. Individuals with AN starve themselves to become underweight, have a fear of gaining weight or getting fat and a skewed body image, resulting in the body being perceived as fat despite being emaciated. The mean duration of AN is approximately 5 years. However, the crossover rate into other eating disorders (EDs) is high, and the mean duration of all aggregated ED episodes, including AN, is estimated to be 10 years [1].

Reduced brain volume in AN was first observed in postmortem studies in the 1950s [2]. The brain atrophy was later corroborated using neuroimaging techniques and the findings included prominent sulci and gyri, enlarged ventricles, and reduced cerebral mass affecting both white and grey matter volume [3, 4]. According to a review from 2018, the volume deficits seem to be largely reversible in remitted adult cases of AN, while the data regarding adolescent AN cases are inconclusive [5].

Low blood pressure and orthostatism are common somatic findings in individuals suffering from both acute and chronic AN. There have been indications that hypotension can result in cerebral hypoperfusion, and lead to an increased long-term risk of developing dementia [6]. The consequences of long-standing hypotension on brain function in individuals with AN have so far not been investigated. Cerebral blood flow studies in individuals with AN have shown that hypoperfusion is localised to certain brain regions. One study on patients with acute AN showed unilateral temporal hypoperfusion, and these findings remained in the two cases that were followed up after weight gain [7]. Another study reported temporoparietal and orbitofrontal hypoperfusion in individuals recovered from AN [8]. Based on the above data, it could be suspected that long-standing hypotension and cerebral hypoperfusion may cause irreversible brain damage in individuals with AN.

The brain volume reductions seen in acutely ill AN patients are more salient in adolescents than in adults [5]. In adolescence, during ongoing brain development, other potential brain-damaging mechanisms, apart from hypotension, must also be considered. An animal study using the activity-based AN model has implied that the reduced brain volume in AN is explained by loss of astrocytes, due to malnutrition [9]. Astrocytes provide neurons with nutrients and play an important role in synaptogenesis [10]. Astrocyte loss could therefore have substantial implications on neuronal function.

Biochemical markers for neuronal injury are used in investigations of dementia and other neurodegenerative disorders. Preferably, these markers are collected from the cerebrospinal fluid, since they probably reflect brain pathology better than markers collected from peripheral blood. However, recent studies indicate that some markers, including neurofilament light chain protein (NfL), can also be valid in serum [11].

Increased plasma NfL levels have been reported in Alzheimer’s disease [12], mild cognitive impairment [12] and traumatic brain injury [13], and represent increased rates of axonal degeneration [14]. There is a strong correlation between cerebrospinal NfL and plasma NfL [15]. In 2019, a Swedish study compared plasma NfL levels in women with current AN (ill for at least 1 year, but the majority consisted of chronic AN cases), women recovered from AN at least 1 year ago, and normal-weight female controls [16]. When adjusted for age, the AN group with current illness exhibited significantly higher plasma levels than both the recovered AN group and healthy controls. BMI correlated negatively with plasma NfL levels across all three groups [16].

We have followed a community-based group of individuals with teenage-onset AN prospectively for 30 years, together with a control group matched for age, schooling and gender. At the 30-year follow-up, the majority was fully recovered from AN and other EDs, but one in five had a chronic ED [1]. In the present study, based on data from the 30-year follow-up, we hypothesised that the individuals with adolescent-onset AN would exhibit increased serum levels of NfL compared with matched controls, indicating that AN has a neurodegenerative impact in the long term. We further hypothesised that serum NfL levels would correlate with AN duration.

## Methods

Fifty-one individuals with teenage-onset AN and 51 healthy individuals have been followed prospectively for 30 years in Gothenburg, Sweden. At the time of the original study in 1985, all students born in 1970, attending the eighth grade and living in Gothenburg, Sweden, were screened for AN. Twenty-four adolescents (22 girls and 2 boys) were assigned an AN diagnosis and constituted a population-based group of adolescent-onset AN. Another 27 AN cases (26 girls and one boy) with adolescent onset, not born in 1970 but in adjacent years, were also discovered at the time of screening and constituted a population screening group. The population-based group and the population screening group exhibited a very similar group structure and were therefore pooled to form a group of 51 individuals (48 girls and 3 boys), the AN group. All individuals met the criteria for AN according to the DSM-III-R [17]. The AN diagnostic criteria were also met according to later versions of the DSM, the DSM-IV from 1994 [18] and the DSM-5 from 2013 [19].

The school health nurses involved in the AN screening in Gothenburg were also asked to select healthy comparison cases matched for schooling, age and sex and without a history of ED. This group consisted of 51 adolescents (48 girls and 3 boys) and will be referred to as the COMP group. For further details, see Råstam et al. [20] and Råstam [21].

The 51 AN cases and 51 COMP cases were assessed at mean ages of 16 (AN Study 1), 21 (AN Study 2), 24 (AN Study 3), 32 (AN Study 4), and 44 years (AN Study 5) [1, 20–24]. After 30 years, at the mean age of 44 years, 98 of the 102 individuals (AN 47; COMP 51; participation rate: 96%) agreed to undergo psychiatric and anthropometric assessments.

### Procedures

All individuals were interviewed regarding current psychiatric disorders using the semi-structured Mini International Neuropsychiatric Interview (MINI 6.0) [25]. We added the ED module of the Structured Clinical Interview for DSM-IV (SCID-I) [26], which contains additional questions regarding AN and binge-eating disorder. The MINI and SCID-I interviews are both based on DSM-IV criteria and we included a checklist for DSM-5 EDs to gather additional information on EDs according to the DSM-5. Three outcome measures were used at the 30-year follow-up: (i) the Global Assessment of Functioning scale (GAF)—a global rating that estimates the degree of psychiatric symptoms and level of functioning; (ii) the Morgan Russell averaged scale score [27]—an established outcome scale for EDs, where weight, dieting, body and shape concerns, menstruation, social functioning, mental and psychosexual status during the last 6 months are summarised into a composite score, and (iii) the 36-item Short Form Health Survey (SF-36) [28]—a health-related quality of life self-report instrument. For further details, see Rydberg Dobrescu et al. [1].

The participants were asked whether they or their parents had been assigned a diagnosis of dementia. The Swedish National Patient Register for in- and outpatient treatment was checked regarding diagnoses of dementia according to the ICD-9 (code 290–294) and the ICD-10 (code F00-F07), and regarding the ICD-10 diagnosis “unspecified symptoms and signs involving cognitive functions and awareness” (code R41.3), and the corresponding ICD-9 diagnosis “memory loss” (code 780.93). The register check was limited to the participants, i.e. parents were not included.

Thirty-four individuals (all females) in the AN and 41 individuals (38 females and three males) in the COMP group agreed to blood samples being collected to assess serum NfL. All individuals were weighed and measured, and their body mass index (BMI) was calculated. Only the females in the two groups (AN *n* = 34; COMP *n* = 38) were included in the study, due to a tendency towards higher CSF NfL concentrations in males compared with females in the general population, which is likely reflected in serum [29].

### Measurement of NfL concentrations

The blood samples were collected during the period May 2015 until November 2016. The samples were processed and frozen at − 80 °C, according to standardised procedures. Serum NfL concentration was measured with an in-house Single molecule array (Simoa) assay (Quanterix®, Billerica, MA), a method delivering a roughly 1000-fold increase in sensitivity (for detailed instructions, see Rohrer et al. [30]). There were no technical issues when analysing the samples. All samples were measured as singlicates with analytical variation monitored using two quality control (QC) samples in the beginning and end of each plate. For a QC sample with an NfL concentration of 44.4 pg/mL, the coefficient of variation (CV) was 4.0%. For a QC sample with an NfL concentration of 138 pg/mL, the CV was 9.0%. Serum NfL is stable at − 80 °C and there is no loss of NfL after at least four freeze–thaw cycles [31].

### Full ED symptom recovery

In accordance with our definition of full ED symptom recovery in AN Study 3 [23] and AN Study 4 [24], we used a modification of the criteria defined by Strober and colleagues [32]. A fully ED-recovered individual must have been free of all criteria of AN, bulimia nervosa or binge-eating disorder for a minimum of 6 consecutive months. The definition further requires no weight deviation, no compensatory behaviours and absence of weight phobia during the last 6 months.

## Ethical approval

This study was approved by the Regional Ethical Review Board at the University of Gothenburg (398-14).

### Statistical analyses

Due to small sample sizes, normal distribution could not be assumed and, for this reason, only non-parametric tests were used. The Mann–Whitney *U* test was applied for analyses with continuous outcome variables (e.g. comparison of serum NfL concentrations between the AN and COMP group, comparison of age between participants and dropouts in the AN and COMP groups). Spearman rank order correlations were used to examine possible associations between serum NfL concentration and other continuous variables, including age, at the 30-year follow-up. Chi-square tests, including Fisher’s exact tests, were performed to analyse differences between groups with regard to categorical outcomes. All significance tests were two-sided and conducted at the 5% significance level.

## Results

The mean ages were 44.2 years in the AN group and 44.3 years in the COMP group. Table 1 shows the anthropometric and demographic data, and current ED diagnoses in the AN and the COMP group, respectively. A dropout analysis regarding participants who declined the collection of blood samples at the 30-year follow-up (AN *n* = 13; COMP *n* = 10) showed no significant differences between participants and dropouts in terms of age, BMI, GAF, Morgan Russell averaged scale score, full ED symptom recovery and AN duration (the last two variables only apply to the AN group) at the 30-year follow-up.

**Table 1:** Anthropometric and demographic data and current eating disorder diagnoses in the AN group and the COMP group

	AN group (*n* = 34)	COMP group (*n* = 38)	*p*
Weight (SD; range)	65.2 (14.8; 45.3–106)	70.5 (14.1; 46.3–104)	0.06
Height (SD; range)	166.1 (6.1; 156–178)	168.1 (6.6; 157–189)	0.33
BMI (SD; range)	23.6 (5.7; 16.3–41.5)	25.0 (5.2; 17.2–39.0)	0.16
Anorexia nervosa	2 (5.9%)	0	
Bulimia nervosa	0	0	
Binge-eating disorder	1 (2.9%)	0	
Other specified feeding or eating disorders	3 (8.8%)^a^	1 (2.4%)^b^	
Any kind of eating disorder	6 (17.6%)	1 (2.6%)	0.047
Full ED symptom recovery^c^	20 (58.8%)	NA	

*AN* anorexia nervosa, *COMP* comparison, *SD* standard deviation, *BMI* body mass index (kg/m^2^), *ED* eating disorder, *NA* not applicable

^a^One case of purging disorder, one case of binge-eating disorder, and one case of atypical anorexia nervosa

^b^One case of night-eating syndrome

^c^A full ED symptom recovered individual must have been free of all criteria of AN, bulimia nervosa or binge-eating disorder for a minimum of 6 consecutive months. The definition further requires no weight deviation, no compensatory behaviours and absence of weight phobia during the last 6 months. BMI was significantly lower in the full ED symptom recovery subgroup compared with the subgroup without full symptom recovery (full recovery: 22.0 kg/m^2^; without full recovery: 25.4 kg/m^2^; *p* = 0.032)

**Fig. 1:**
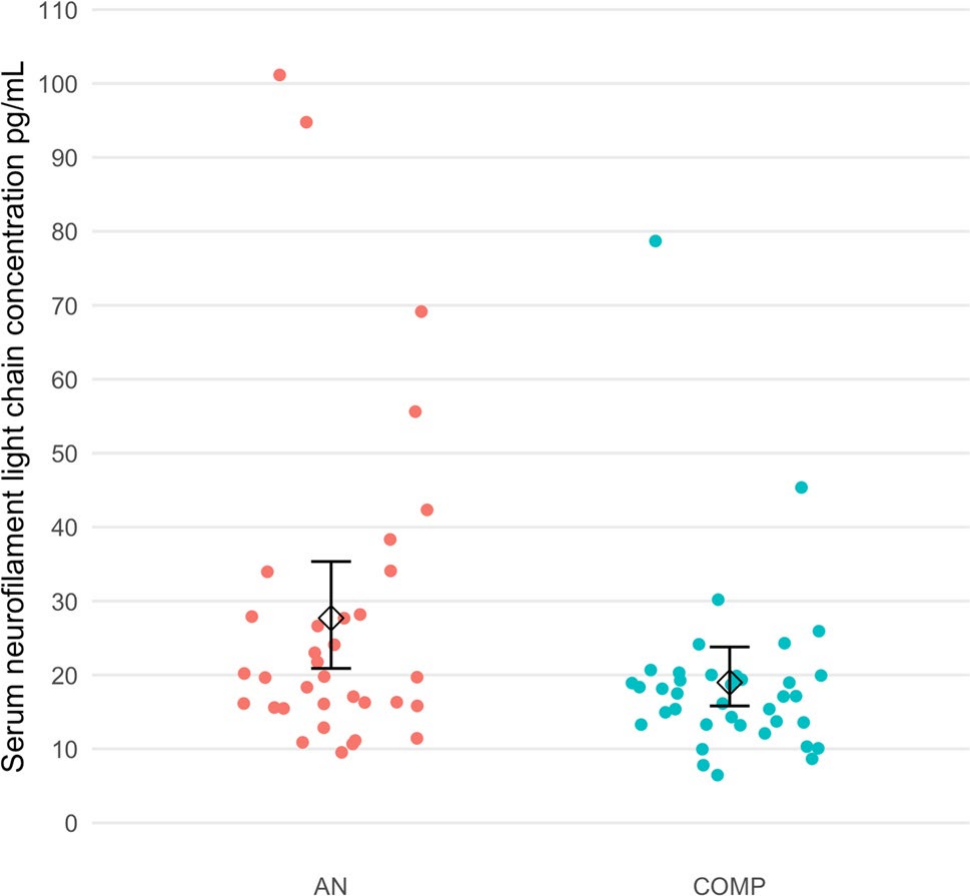
Serum neurofilament light chain concentration in individuals in the AN and COMP groups. *AN* anorexia nervosa, *COMP* comparison; the whiskers represent 95% confidence intervals

The serum NfL concentrations were significantly higher in the AN group compared with the COMP group (*p* = 0.041) (Table 2; Fig. 1). Serum NfL concentrations did not differ between those who showed full ED symptom recovery and those who did not in the AN group (*p* = 0.42). There was no difference in mean serum NfL concentrations between those with a current ED diagnosis and those without an ED in the AN group (current ED 32.1 pg/ml; median 18.1; SD 23.9; no current ED 26.7 pg/ml; median 20.0; SD 21.9; *p* = 0.74).

**Table 2:** Serum neurofilament light chain protein data in AN and COMP group, and in subgroups of individuals without neurological/autoimmune disorders, without current eating disorder, without current psychotropic medication and with full eating disorder symptom recovery, respectively

	AN group (*n* = 34)	COMP group (*n* = 38)	*p*
Serum NfL (pg/ml) (SD)	27.7 (22.0)	19.0 (12.1)	0.041
Serum NfL (pg/ml) in individuals without a neurological or autoimmune disorder or dementia workup (SD)	27.4^a^ (23.0)	18.8^b^ (12.4)	0.060
Serum NfL (pg/ml) in individuals without a current ED (SD)	26.7^c^ (21.9)	19.0^d^ (12.3)	0.054
Serum NfL (pg/ml) in individuals without current psychotropic medication (SD)	28.5^e^ (22.1)	18.6^f^ (12.3)	0.042
Serum NfL (pg/ml) in full ED symptom recovered individuals (SD)	29.5^g^ (24.8)	N/A	

A full ED symptom recovered individual must have been free of all criteria of AN, bulimia nervosa or binge-eating disorder for a minimum of 6 consecutive months. The definition further requires no weight deviation, no compensatory behaviours and absence of weight phobia during the last 6 months

*AN* anorexia nervosa, *COMP* comparison, *NfL* Neurofilament light chain protein, *SD* standard deviation, *ED* eating disorder, *N/A* not applicable

^a^Based on 29 individuals

^b^Based on 36 individuals

^c^Based on 28 individuals

^d^Based on 37 individuals

^e^Based on 22 individuals

^f^Based on 36 individuals

^g^Based on 20 individuals

One woman in the AN group had undergone a dementia workup due to problems with her memory but had not been assigned a diagnosis (serum NfL concentration: 34.1 pg/ml). No individuals in the AN or COMP group had received a diagnosis of dementia, memory loss (ICD-9) or unspecified symptoms and signs involving cognitive functions and awareness (ICD-10), according to the register search of in- and outpatient treatment. Four women in the AN and three women in the COMP group reported having a parent with diagnosed dementia. Serum NfL concentrations were not higher among those with a parent with dementia, neither in the AN nor in the COMP group (AN *p* = 0.82; COMP *p* = 0.61).

### Correlation between serum NfL concentration and other variables

**Fig. 2:**
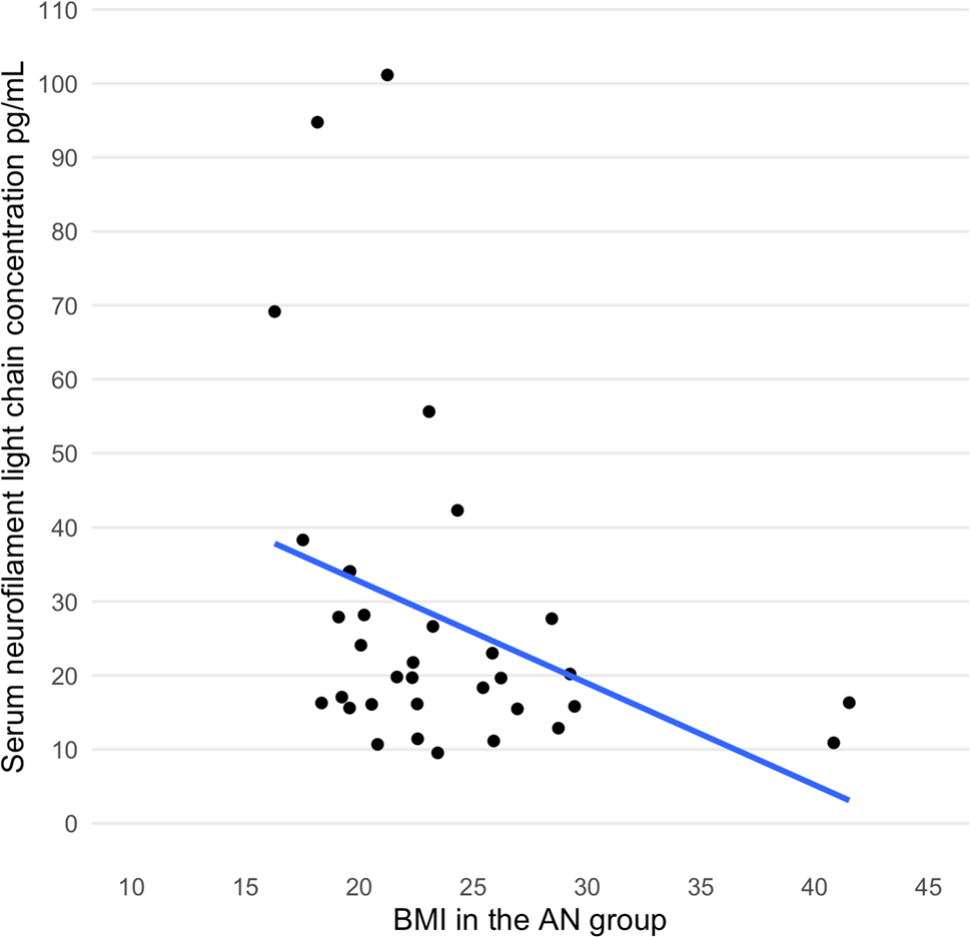
Correlation between BMI and serum neurofilament light chain concentration in individuals in the AN group. *BMI* body mass index, *AN* anorexia nervosa

There was a significant negative correlation between serum NfL concentration and current BMI in the AN but not in the COMP group (AN *r* = −0.44 COMP *r* = −0.22) (Fig. 2). Age at the 30-year follow-up did not correlate significantly with the serum NfL concentration in the two groups (AN *r* = 0.22; COMP *r* = 0.12). There was no significant correlation between serum NfL and GAF (AN *r* = 0.050; COMP *r* = −0.047), Morgan Russell averaged scale score (AN *r* = 0.13; COMP *r* = 0.10), AN duration, including all episodes (AN *r* = 0.15; only applies to the AN group), ED duration, including all episodes (AN *r* = 0.038; only applies to the AN group), or lowest lifetime BMI (based on data from mean age 32 years; AN Study 4) (AN *r* = −0.060; only applies to the AN group). Nine individuals in the AN group and two individuals in the COMP group were currently using psychotropic medication (antidepressants, in most cases). Neither the AN nor the COMP group showed any differences in NfL concentration between those who currently used psychotropic medication and those who did not (AN *p* = 0.75; COMP *p* = 0.085).

There was a tendency towards higher mean serum NfL concentrations among the individuals without a current ED in the AN group compared with the COMP group (*p* = 0.054) (Table 2). Among the individuals without a current ED, there was no significant correlation between serum NfL concentrations and AN duration, including all episodes (AN *r* = 0.061; only applies to the AN group), or ED duration, including all episodes (AN *r* = −0.059; only applies to the AN group).

We checked the national patient register for in- and outpatient treatment to exclude medical and neurological disorders that could potentially affect serum NfL concentrations. There were four women in the AN group with medical/neurological disorders (type 1 diabetes with “neurological complications”, angina + migraine, migraine, and Crohn’s disease). In the COMP group, two women had medical/neurological disorders (epilepsy, and migraine). One woman with registered medical/neurological disorder in each group had serum NfL concentrations above the 95% confidence interval of their group. When cases with medical/neurological disorders and dementia workup were excluded, the serum NfL concentrations still tended to be higher, but not significantly higher, in the AN group compared with the COMP group (*p* = 0.060) (Table 2).

## Discussion

To our knowledge, this is the first paper on a neuronal injury biomarker at long-term follow-up of AN. Thirty years after adolescent-onset AN, at a mean age of 44 years, we found significantly higher serum NfL concentrations in the AN group compared with the COMP group (*p* = 0.041). A trend (albeit statistically non-significant, *p* = 0.060) towards increased serum NfL concentrations in the AN group remained when participants with medical/neurological disorders were excluded from the two groups. There was no correlation between serum NfL concentration and duration of AN or age at the 30-year follow-up. A negative correlation was found between serum NfL concentration and current BMI in the AN group. No individuals in the AN or the COMP group had been assigned a diagnosis of dementia.

One previous study has investigated serum NfL concentrations in AN. The study included individuals with ongoing AN, recovered AN and healthy controls with a mean age between 26 and 31 years [16]. After adjusting for age, significantly higher NfL concentrations were found in individuals with current AN compared with recovered AN cases and controls. The age at sampling correlated positively with the NfL concentration. The present long-term follow-up study shows results in line with the study mentioned above, i.e. serum NfL concentration was higher in the AN group than in the COMP group. However, our study comprised few individuals with ongoing ED. In addition, serum NfL concentration did not differ between those with a current ED and those without an ED in the AN group. The study by Nilsson et al. [16] also differed from the present study in terms of mean ages, between 26 and 31 years, compared with the current study representing a middle-aged group with a mean age of 44 years. Our median NfL data in the AN group (19.7 pg/ml) should be compared primarily with the two recovered AN samples in Nilsson et al., with median NfL levels of 9.2 and 11.1 pg/ml, respectively. Our COMP group also had a higher median serum NfL concentration (17.3 pg/ml) compared with the control groups in the publication by Nilsson et al. (7.8 pg/ml and 9.3 pg/ml). The higher median serum NfL levels in the 30-year follow-up study can probably be explained by the higher mean age in our group [16]. Nilsson et al. did not find higher serum NfL concentration in their recovered AN groups compared with their control groups [16]. In the present study, when individuals with a current ED were excluded, we found a statistically non-significant trend towards higher serum NfL concentrations in the AN compared with the COMP group (*p* = 0.054).

The increased serum NfL concentrations in our AN group should be interpreted with caution. When excluding individuals with current neurological/medical disorders, there was only a tendency towards higher serum NfL concentrations in the AN compared with the COMP group. Ehrlich’s group has twice reported negative findings regarding the GFAP and S100B brain damage markers in peripheral blood in individuals with acute AN [33, 34]. GFAP is a monomeric intermediate filament protein found in the astroglial skeleton in white and grey matter and is regarded as brain specific. It is considered a marker for various types of brain damage, including traumatic brain injury [35] and neurodegenerative disorders [36]. An increased concentration of S100B in peripheral blood, a protein also abundant in astroglia, signals brain tissue damage, including hypoxia and traumatic brain injury [34]. Our data, as opposed to those from the studies of Ehrlich et al*.*, did not mirror the acute AN phase. Some individuals in the present study still had an ED. There was no significant difference regarding NfL levels between those with and those without a current ED. Forthcoming studies should use larger samples to increase power to detect potential differences. Our hypothesis that a longer AN duration would result in higher serum NfL levels was not confirmed. This finding weakens the reasoning that long-standing AN could be deleterious to the brain. However, NfL only reflects axonal degeneration. Other studies have reported neurohistological abnormalities in individuals with AN and in AN animal models indicating degeneration associated with “pseudoatrophia cerebri”. A postmortem study showed quantitative and qualitative changes in neuronal dendritic spine morphology [37], and animal studies using dehydration-induced, forced food-restricted, or activity-based AN models have found reduced astrocytes in the hippocampus, corpus callosum, and cerebral cortex [9, 38–40]. Future studies need to collect biomarkers from cerebral spinal fluid to investigate possible degenerative processes caused by AN in greater detail.

### Strengths and limitations

This study has several strengths. The individuals in the AN and the COMP group have been followed prospectively for almost 30 years. The sample is community based, which results in a more naturalistic investigation of AN over several decades compared with clinically based studies, which cannot claim representativeness. We have had the privilege of access to a huge amount of data from the five examinations that have been performed over the years. This has made it possible to consider associations between the NfL biomarker and other variables over a 30-year time span.

There are some limitations to the present study. Firstly, no brain imaging was performed and therefore we have no information on the occurrence and extent of brain atrophy. Assessments of brain volume, including estimates of grey and white matter volumes separately and correlated to serum NfL data, would have been very informative. Secondly, we did not perform any psychometric tests pertaining to cognitive ability or signs of dementia, including the Mini Mental State Examination [41]. Thirdly, the sample size was relatively small, yet we found a statistically significant difference in serum NfL concentrations between the AN and the COMP group. Fourthly, the present study was cross-sectional in terms of serum NfL data, and therefore we cannot draw any conclusions regarding the NfL trajectories in the AN and COMP groups over time.

## Conclusions

Thirty years after AN onset, when most individuals had full ED symptom recovery, we found increased serum NfL concentration, indicating higher rates of axonal degeneration, also seen in Alzheimer’s disease and other neurodegenerative disorders, in the AN compared with a control group. These findings need to be replicated, since some of the differences between the AN and the COMP group failed to reach statistical significance (*p* = 0.060). However, our results could pave the way for a new research area regarding very long-term consequences of AN. Future prospective follow-up studies of AN should include repeated examinations of biomarkers for brain health to deepen the understanding of neurodegeneration as a possible long-term consequence of AN.
